# Construction and practice of a comprehensive nursing skills course with simulation in an RN-BSN program in China: a quasi-experimental study

**DOI:** 10.1186/s12909-021-02998-w

**Published:** 2022-01-03

**Authors:** Yuanyuan Zhu, Aihong Wang, Yamei Bai, Min Xu, Haiyan Yin, Qinyi Gao

**Affiliations:** 1 School of Nursing, Nanjing University of Chinese Medicine, 138 Xianlin Avenue, Nanjing, 210023 Jiangsu Province China; 2 Nursing Department, Nanjing Jiangning Hospital, 169 Hushan Road, Nanjing, 211100 Jiangsu Province China

**Keywords:** Nursing education, Simulation, RN-BSN program, Comprehensive nursing skills

## Abstract

**Background:**

Simulation has been widely used in the teaching of pre-licensed nursing students and has shown positive results. However, there is limited evidence regarding the application of a comprehensive nursing course with simulation for Associated Degree in Nursing (ADN)-prepared nurses with different work experience times. Therefore, this study aimed to evaluate the satisfaction, self-confidence, and perceptions of scenario simulation among Chinese nurses in a comprehensive nursing skills course in an RN-BSN program.

**Methods:**

A single-group post-test approach was used in the current study. Participants that completed a comprehensive nursing skills course with simulation in an RN-BSN program were enrolled.

**Results:**

The mean satisfaction, self-confidence, educational practice, and simulation designs scores were rated highly. Self-confidence (*p* = 0.002), active learning (*p* = 0.020), collaboration (*p* = 0.030), support (*p* = 0.008), and problem-solving (*p* = 0.007) were significantly higher among students with more work experience compared to those with less experience. Then, four themes were analyzed: enthusiasm for learning, ability to experience different feelings during role-play, hybrid teaching format, and simulation fidelity.

**Conclusions:**

Results demonstrated that a comprehensive nursing skills course with simulation might improve Chinese ADN-prepared nurses’ satisfaction and self-confidence in learning. Nurses with work experience gave a high rate to the scenario simulation, demonstrating that simulation can be widely applicable for students with different characteristics. Finally, the teaching strategy in the present study can be applied to more RN-BSN programs.

## Background

An evidence-based recommendation from the National Academy of Medicine (NAM) states that 80% of registered nurses (RNs) in the United States should possess a Bachelor of Science in Nursing (BSN) by 2020 [1]. The NAM’s goal to increase the number of BSNs in the United States is based on previous studies which have indicated that a higher proportion of BSNs offering care during hospital stays might lead to better patients outcomes [2, 3]. Currently, more than 3.2 million RNs are registered in China. Moreover, a survey of 51,406 RNs, conducted in 2015 in China, revealed that 94.8% of them received a college education or above and 53.8% had bachelor’s degrees [4]. The National Health Commission of the People’s Republic of China has indicated that the educational mobility of RNs is constantly improving in China, but Associated Degree in Nursing (ADN)-prepared RNs are still important in clinical nursing teams [5].

RN-BSN programs are the main way to improve nurses’ educational backgrounds. However, nurses have expressed their fear and lack of self-confidence when considering an RN-BSN program. Fear has been described to be related to a previous negative ADN or diploma educational experience, leading nurses to be afraid of going through it again [6]. Additionally, if the RN-BSN program content and methods repeat courses and topics already learned, BSN-RN students might experience a lack of enthusiasm with the learning process [7]. Students who participate in RN-BSN programs would like to master comprehensive care and combine their new knowledge with previous work experiences to find solutions to clinical issues [8]. Therefore, a well-designed comprehensive course should integrate RN-BSN programs.

The increasing demands for teamwork, communication, and critical thinking of nurses led to the appearance of innovative teaching methods in nursing education, including simulation, which is already used as an effective education method. Jeffries defined simulation as “activities that mimic the reality of a clinical environment and are designed to demonstrate procedures, decision-making, and critical thinking through techniques such as role-playing and the use of devices such as interactive videos or mannequins.” Five major components – teacher, student, educational practices, outcomes, and simulation design characteristics – are included in this simulation model [9]. Simulation-based education can be an effective strategy to improve students’ knowledge, skills, behaviors, and satisfaction [10]. Most simulation-based education aims at pre-licensed nursing students, who are different from ADN-prepared nurses. Compared with undergraduate students, most ADN-prepared nurses have full- or part-time work experiences. A Malaysian study found that the work experience duration affected educational interventions effects [11]. Thus, the work experience of ADN-prepared nurses might be a significant variable in simulation education. However, few studies have included simulation in BSN-RN programs for ADN-prepared nurses, in both China and abroad, and the impact of work experience on simulation training has not been clearly explained. Therefore, this study aimed to examine Chinese nursing students’ satisfaction, self-confidence, and perceptions regarding simulation in a comprehensive nursing skills course in an RN-BSN program. Three questions guided this study: (1) What are RN-BSN students’ satisfaction, self-confidence, and perceptions of simulation? (2) Are there any differences in satisfaction, self-confidence, and perceptions between students with different work experience times? (3) What are the students’ opinions about the comprehensive nursing skills course?

## Methods

### Study design

This single-group post-test only designed study was conducted between October and December 2019 to measure students’ satisfaction, self-confidence, and perceptions of simulation in the comprehensive nursing skills course of an RN-BSN program.

### Participants and setting

The participants enrolled in the current study were from an RN-BSN diploma degree studying program in an eastern China university. All participants were working as RNs in medical institutions and all nursing students currently enrolled in the course were invited to participate in the study. Also, grades were not affected by participation in this study. A total of 98 students volunteered to participate, but eight did not complete all surveys. Therefore, the study was completed with 91 (92.9%) students. Written informed consent was obtained and the study was approved by the Ethics Committee of the Nanjing Jiangning Hospital (No. 2019–03-025-K01).

### Intervention

First, researchers reviewed the literature, books, and electronic databases, and summarized the initial draft. Then, five experts were selected to review the draft and made revision suggestions. A modified Delphi Method was used to achieve consensus in two rounds by surveying 12 nursing and clinical nursing experts. After researchers revised the content, based on the panelist’s suggestions, the content was discussed and revised repeatedly in meetings to form the final course. The Scale-level Content Validity Index (S-CVI) was 0.89.

The course included 27 class hours (1 class hour = 40 min) with 7 classes, and each class had 3 to 5 class hours. The course consisted of four modules: nursing education progresses (Module 1), case study (Module 2), scenario simulation (Module 3), and clinical nursing case sharing (Module 4) (Table 1). The scenario simulation module was conducted in a laboratory and the others in the classroom. Module 1 included course schedule introduction, teaching plans, and score composition. The lecture on current nursing education progresses contained case-based learning, team-based learning, scenario simulation, and the combination of online and offline. In module 2, the teacher shared a comprehensive clinical case that provided the patient basic condition, then the students formulated nursing process according to the disease progress. Student-centered group discussion was used to analyze and extend the case. Module 3 was the core component of the course and consisted of theoretical thinking and practical training. The four scenario simulation cases followed the NLN/Jeffries Simulation Framework and were used in each class [9]. To increase the module’s comprehensiveness, medical nursing, surgical nursing, fundamental nursing, health evaluation, nursing interpersonal communication, and health education topics were included. Finally, four sample patient cases of type 2 diabetes, protrusion of lumbar intervertebral disc, chronic obstructive pulmonary disease, and cardiac failure were compiled for scenario simulation. Each scenario simulation contained three phases:

1. Preparation: at the beginning of each simulation class, the teacher introduced the simulation case and provided supplemental theoretical knowledge to help students. Each case provided health records and disease progresses. A short group discussion was adopted to address questions before simulation.
2. Simulation: students were divided into two independent classes, and each class was divided into 6 groups (7 to 8 students per group). Then, students chose between the nurse, patient, family member, or observer roles. According to the characteristics of each case, the nursing process, health education, and discharge instructions were designed for students to practice. The teacher gave students clues to prompt them to take action and students were encouraged to engage in the scenario and role play. Nursing interventions such as urethral catheterization, intramuscular injection, and venous transfusion were practiced on a medical simulator. The scenario simulation template is presented in Table 2.
3. Debriefing: After the role-play, the students and teacher attended a debriefing to review and discuss their feelings and performances.

**Table 1:** Comprehensive nursing skills training

Module	Stage	Subject	Content	Pedagogical approach	Class hour	Setting
Module 1	Preparation	Nursing education progresses	∙ Introduce the course schedule ∙ Learn new pedagogical methods and evaluation mode	∙ Lecture	3	Classroom
Module 2	Analysis	Case study	∙ Analyze clinical case ∙ Discuss the case in group	∙ Student-centered group study; ∙ Case-based learning	3	Classroom
Module 3	Simulation	Scenario simulation	∙ Four cases: type 2 diabetes; protrusion of lumbar intervertebral disc; chronic obstructive pulmonary disease; cardiac failure ∙ Students are divided into 2 classes; each class is divided into 6 groups ∙ Preparation phase; simulation phase; debriefing phase	∙ Student-centered group study; ∙ Role-play in scenario simulation	17	Laboratory; classroom
Module 4	Sharing	Clinical nursing case sharing	∙ Students collect clinical case and make PowerPoint presentations using nursing process in advance ∙ Share case in the class in 16 groups	∙ Presentation by students ∙ Question & Answer session	4	Classroom

**Table 2:** Scenario simulation template: Type 2 diabetes

Initial Scenario	Scenario Progressions	Expected Actions
Xing Chen, male, 72 years old, widowed, living with his daughter. He is admitted to the hospital with due to thirst, excessive drinking for more than 2 years and diagnosed with type 2 diabetes. The symptoms are ignored and not treated before. He doesn’t know much about the disease and is very anxious.	Medical prescription: Glargine insulin 10 U H qn. Metformin 0.5 g po tid. Pancreatic kininogenase 40 U IM qd.	∙ Fundamental nursing: administer intramuscular and subcutaneous injections and dispense oral medications. ∙ Medical nursing & Psychological nursing: explain the effects of medications to patients; comfort the patient’s emotions.
	The 5th day of admission, at 8 p.m., the patient developed dizziness, oppression in chest, and sweating. The patient is very scared.	∙ Medical nursing: recognize the symptoms of hypoglycemia. ∙ Health evaluation: measure blood sugar and vital signs. ∙ Psychological nursing: comfort the patient’s fear.
	Blood glucose is 3.4 mmol/L; T36.4 °C; P94; R22; BP115/75 mmHg.	∙ Fundamental nursing: prepare glucose solution for patients to correct hypoglycemia. ∙ Health education & Nursing interpersonal communication: symptoms, causes, preventions and emergency treatment of hypoglycemia. ∙ Fundamental nursing: retest blood glucose after 15 min.
	Patient is gradually taking diabetes seriously, but is still unclear about how to manage diet and exercise, and feel anxious and confused.	∙ Psychological nursing: reassure and communicate with anxious patient. ∙ Health education & Medical nursing & Communication: teach the patient to manage diet and exercise.
	The patient is ready to be discharged from the hospital tomorrow with medical prescription: Glargine insulin 12 U H qn; However, patient and his daughter don’t know how to inject insulin at home.	∙ Fundamental nursing & Health education & Communication: teach the patient and his daughter methods and precautions for insulin injections at home.

In the last module, students collected real clinical cases and prepared PowerPoint presentations in groups before class. The presentation should reflect the disease progress and the corresponding nursing process. Every group shared their case reports, and a question-and-answer session was performed when required.

### Measurements

After the simulation, a demographic questionnaire was used to collect information regarding age, gender, work unit, and work experience. This study used three scales to measure quantitative outcomes: Students’ Satisfaction and Self-Confidence in Learning, Educational Practices Questionnaire, and Simulation Design Scale. All scales were developed by NLN on a 5-point Likert scale (1 = strongly disagree, 2 = disagree, 3 = undecided, 4 = agree, 5 = strongly agree) [12].

Students’ Satisfaction and Self-Confidence in Learning contained 13 items on a 5-point Likert scale, with two subscales to measure satisfaction (5 items) and self-confidence (8 items) in learning. Higher scores represented greater satisfaction and self-confidence levels. The Cronbach’s alpha values of the Chinese version of these two scales were 0.87 and 0.83, respectively [12].

The Educational Practices Questionnaire contained 16 items to evaluate students’ views of educational practices in the simulation. Ten items measured active learning, and two were designed to examine collaboration, diverse ways of learning, and high expectations. The Cronbach’s alpha value of the Chinese version of the scale was 0.90 [12].

The Simulation Design Scale involved five simulation design features: objectives/information (5 items), support (4 items), problem-solving (5 items), feedback (4 items), and fidelity (2 items). This scale was used to evaluate the students’ perceptions of the design. The Cronbach’s alpha value of the Chinese version of the scale was 0.87 [12].

To gain an in-depth understanding of students’ perceptions of and suggestions for the course, two open-ended survey questions were included at the end of the questionnaire: (1) What did you learn from the course? (2) What are your suggestions for the course?

### Data collection and analyses

All students who participated in the study provided informed consent. The demographic data, scales, and open-ended questions were collected at the end of the last class. In mainland China, the *Training Outline for Newly Recruited Nurses* was developed in 2016 and provides detailed regulations on training methods, time, content, and assessment [13]. Every nursing student who graduates from school and begins to work in the hospital should participate in this training. The training content includes basic theoretical knowledge, basic operation, professional theory, and practical ability to improve nurses’ clinical competence. The training lasts 2 years, but each province or city has its specific arrangement. In Nanjing, Jiangsu Province, the training period for new nurses is 3 years. Only when the nurse has passed the assessment the training can be completed. Nurses who have accomplished the three-year training are considered experienced. Most participants in this study worked in Nanjing’s hospitals, thus 3 years was used as the cutoff point to judge work experience.

Data were analyzed using IBM SPSS 22.0. Means, standard deviations (SDs), frequencies, and percentages were used for descriptive statistics. The homogeneity of demographic characteristics between the two groups was tested with the independent *t*-test and the χ^2^ test. Differences in the three scales between groups were analyzed by the independent *t*-test. A *p* < 0.05 was considered statistically significant. The two open-ended questions were independently analyzed by two researchers using thematic analysis. Each researcher identified themes, which were reviewed and generated.

## Results

### Demographic characteristics of the participants

Ninety-one participants (93.4% female) were included in this study, with an average age of 25.16 years (SD = 2.52). They had graduated from college and obtained RN certificates, and 54.9% were working in tertiary hospitals in China. The median work experience time was 3 years. Participants were divided into two groups based on work experience times: less work experience (< 3 years) and more work experience (≥ 3 years) (Table 3).

**Table 3:** Demographic characteristics of participants

Variables	Total (***n*** = 91) M ± SD or n (%)	Less work experience (< 3 years) (***n*** = 38) M ± SD or n (%)	More work experience (≥ 3 years) (***n*** = 53) M ± SD or n (%)	χ2 or ***t***	***p***-Value
Age (years)	25.16 ± 2.52	23.63 ± 1.51	26.26 ± 2.53	−6.189	< 0.001
Gender					
Male	6 (6.6)	3 (7.9)	3 (5.7)	< 0.001	0.999
Female	85 (93.4)	35 (92.1)	50 (94.3)		
Work unit					
Tertiary hospital	50 (54.9)	26 (68.4)	24 (45.3)	4.990	0.164
Secondary hospital	14 (15.4)	4 (10.5)	10 (18.9)		
First hospital	5 (5.5)	2 (5.3)	3 (5.7)		
Others	22 (24.2)	6 (15.8)	16 (30.1)		
Professional title					
Nurse	72 (79.1)	28 (100.0)	44 (30.2)	1.167	0.280
Nurse practitioner	19 (20.9)	0 (0.0)	19 (17.0)		

*M* mean, *SD* standard deviation

### Students’ satisfaction and self-confidence in learning with different work experience times

As shown in Table 4, students’ satisfaction (4.31 ± 0.57) and self-confidence (4.22 ± 0.49) were high with the simulation. Self-confidence was significantly higher in students with more work experience (*t* = − 3.244, *p* = 0.002), whereas no significant differences were found for satisfaction.

**Table 4:** Jefferies simulation scale in students with different work experience

Scale	Total M ± SD	Less work experience (< 3 years) M ± SD	More work experience (≥ 3 years) M ± SD	Mean difference	***t***	***p***-Value
Students’ Satisfaction and Self-confidence in Learning						
Satisfaction with current learning	4.31 ± 0.57	4.37 ± 0.58	4.28 ± 0.56	0.093	0.771	0.443
Self-confidence in learning	4.22 ± 0.49	4.03 ± 0.46	4.35 ± 0.46	−0.319	−3.244	0.002
Educational Practices Questionnaire						
Active learning	4.15 ± 0.46	4.02 ± 0.49	4.25 ± 0.42	− 0.227	−2.360	0.020
Collaboration	4.32 ± 0.50	4.18 ± 0.53	4.42 ± 0.47	− 0.231	−2.206	0.030
Diverse ways of learning	4.34 ± 0.51	4.22 ± 0.54	4.42 ± 0.48	−0.191	−1.782	0.078
High expectations	4.35 ± 0.48	4.39 ± 0.50	4.31 ± 0.47	0.083	0.814	0.418
Simulation Design Scale						
Objectives and information	4.24 ± 0.52	4.17 ± 0.57	4.29 ± 0.48	−0.118	−0.172	0.286
Support	4.24 ± 0.53	4.09 ± 0.54	4.34 ± 0.50	− 0.273	−2.724	0.008
Problem-solving	4.28 ± 0.49	4.12 ± 0.45	4.39 ± 0.48	−0.273	−2.751	0.007
Feedback	4.29 ± 0.51	4.19 ± 0.57	4.35 ± 0.47	− 0.163	− 1.505	0.136
Fidelity	3.96 ± 0.57	3.91 ± 0.59	3.99 ± 0.56	−0.083	− 0.679	0.499

*M* mean, *SD* standard deviation

### Educational practices questionnaire with different work experience times

The scores of subscales were above 4. Active learning (*t* = − 2.360, *p* = 0.020) and collaboration (*t* = − 2.206, *p* = 0.030) were higher in students with more work experience and significant differences were found between groups. Diverse ways of learning and high expectations were not significantly affected by work experience time (Table 4).

### Simulation design scale with different work experience times

The highest mean score was 4.29 for feedback, and the lowest was 3.96 for fidelity. The mean support score for students with less work experience was lower than those with more work experience (*t* = − 2.724, *p* = 0.008). Also, a similar result was found for problem solving (*t* = − 2.751, *p* = 0.007) (Table 4).

### Open-ended questions

Question 1: What did you learn from the course?

Sixty-two answers were collected for this question. Most students were satisfied with teaching efficiency and two themes emerged:

- Theme 1: Enthusiasm for learning.

Most nursing students noted that the organization of this course was different from a traditional teaching model. The form was novel, and the content was comprehensive and close to clinical practice, which can greatly enhance nursing students’ learning enthusiasm and initiative, as shown by their comments.

*“The content of the course was pretty new, involving multiple modules, not simply repeating the learned knowledge, each of which had a corresponding training goal. The comprehensive process let me think more critically.”*

*“Different from the previous learning mode, the scenario simulation module was very interesting. Everyone was assigned a task and engaged in the simulation, which could improve participation and enthusiasm.”*

- Theme 2: Ability to experience different feelings during the role-play.

Role-playing as patients or family members enables further understanding of their emotions and situation. When acting as an observer, students had an opportunity to catch problems that were not as easy to notice at work.

*“In the scenario simulation, I also tried to play other roles. This role transition let me learn to think differently and better understand patient’s feelings. Once I was simulated as an observer, standing in the perspective of an outsider. This allowed me to discover some problems that I didn't notice in my work.”*

Question 2: What are your suggestions for the course?

Of the 54 responses to this question, two themes emerged:

- Theme 1: A hybrid teaching format.

Since all the nursing students participating in this course had full-time jobs, they had less spare time, which sometimes caused scheduling inconveniences. Therefore, a common suggestion was to try hybrid teaching.

*“The teaching method could adopt online and offline mixed learning; the theoretical courses could use online learning, and the simulation module could be concentrated on offline teaching.”*

- Theme 2: Simulation fidelity.

The simulation fidelity in the course was not high, which may have affected teaching effectiveness. Higher fidelity would increase the simulation authenticity.

*“The fidelity of the medical simulator was a little low. There was no way to demonstrate the changes of the disease, and it would be better to use high-fidelity simulation.”*

## Discussion

This comprehensive nursing skills course consisted of four modules, included multiple teaching methods, and was provided for RN-BSN students over 3 months. Students reported their perceptions, satisfaction, and self-confidence regarding the scenario simulations in the course.

To improve the applicability of the course and the learning interest of students, we designed a comprehensive course that integrates several nursing courses to train multiple abilities. Previous studies that included simulation training were directed to certain courses and specific populations, such as undergraduate nursing students, emergency nurses, and midwives [14]. However, the students in this study came from multiple departments of different hospitals and had previous work experience. Therefore, a comprehensive curriculum is more suitable for them. Considering students’ theoretical foundations, we designed more interactive sessions to increase participation. Finally, results indicated that students were satisfied with the course.

In this study, students reported positive results on satisfaction and self-confidence regarding the simulation. These findings showed that students’ satisfaction and self-confidence were at a high level. Compared with students with less work experience, those with more experience showed significantly higher self-confidence in the simulation (*p* = 0.002). Previously, APN students who had work experience had higher self-confidence scores than those without, but the difference was not significant [15]. It is not surprising that students with more work experience feel more confident. Nurses accumulate work experience in dealing with clinical problems, which makes them more comfortable with simulation training. Students’ self-confidence in learning might be related to their basic knowledge and experience. Therefore, previous work experiences should be considered when students’ self-confidence in learning is evaluated.

Active learning is a necessary element in the best educational practices. Traditional educational models are teacher-centered and courses for RN-BSN students should be learner-centered instead [16]. Previously, it has been observed that simulation should be designed considering students’ needs and feelings, and students must be active participants [17]. Active learning is one of the variables in NLN/Jeffries, and role play in interactive learning environments contributes to students’ engagement in the learning process [9]. Most students agreed that active learning was used sufficiently in the simulation process. Additionally, a higher score for students with more work experience was detected (*p* = 0.020). Satisfaction and active learning score were also higher among students with more work experience. A recent study showed that active learning was related to self-confidence [18], which might explain the above results.

Healthcare environments are complex and dynamic. Efficient teamwork and communication among healthcare professionals are necessary to meet the needs of different patients [19]. Collaborations occur throughout the entire nursing process. Nurses work not only with nursing colleagues and staff but also with patients and their family members. In this study, different roles and scenarios were prepared in the simulation to enhance students’ collaborative skills and consciousness. Simulation has been described as an appropriate methodology to improve collaborative skills for pre-licensed nursing students [20]. Although the population of that last study was different, in the present one, collaboration was recognized by participants. A significant difference was found for collaboration in more work-experienced students compared to those with less experience (*p* = 0.030). For novice nurses, being able to complete nursing tasks is the first step, but tacit teamwork is a more difficult task. Compared with novice nurses, experienced ones are more able to cope with teamwork tasks and are more confident during this process [21]. They understand better their role in the team and how to cooperate and communicate. Teamwork is common in clinical practice, and team support and collaboration capabilities can also affect efficiency [22]. In simulation training, researchers could set teamwork to help students improve awareness, communication, and leadership in collaboration.

In the Simulation Design Scale, the scores were greater than 4, except for fidelity. Most students felt supported in the simulation since the teacher helped them become familiar with the case by sharing and explaining the case before the simulation. During the simulation, the teacher gave instructions and encouragement to students and was available to answer questions at any time. Additionally, although students were encouraged to think independently, they could choose to work in groups to discuss and address any questions. Therefore, support came from the teacher and their peers. Results revealed a significant difference in the support subscale for students with different work experience times (*p* = 0.008). These results might be explained by the greater proactivity of more work-experienced students in their interactions and more willing to ask questions.

Problem-solving is an essential ability for nurses. Therefore, appropriate problem-solving complexity is very important in the design of the simulation [23]. The mean problem-solving score was 4.28, indicating that students agreed with this statement. Also, a significant difference was detected between students with different work experience times (*p* = 0.007) (Table 4). These results are similar to a previous American study [15]. In the current study, the simulation was designed based on the clinical nursing process and basic nursing skills ranging in difficulty from simple to complex. This progression model would help students improve their problem-solving skills over time. The highest mean subscale score was feedback, consistent with Lubber’s research [24]. Debriefing might include feedback to learners [25] and can be a helpful, informative, and encouraging step in the simulation, providing students with opportunities to review and talk about their performance. In the present study, debriefing was student-centered, encouraging students to evaluate their performance and share their feelings after the simulation. This process can help students to take a fresh look at their strengths and weaknesses. Moreover, it is a good chance to reflect on their daily nursing care. A systematic review showed that the form, time, whether to use structured guidelines and media will affect the debriefing and simulation training effects [26]. Therefore, to achieve the desired effect, researchers should pay more attention to these factors when designing debriefing.

Finally, two open-ended questions were used for supplemental overall assessment and suggestions for the course. Students gave positive comments on each module and said that the comprehensive course was rich in content and improved their participation and learning motivation. However, students also made improvement suggestions. Some students mentioned an issue related to inconvenient time and place. Therefore, an online and offline hybrid approach can be considered in further studies. In the present study, fidelity scored lowest in the Simulation Design Scale, indicating that fidelity should be regulated to an adequate level for RN-BSN students. Cost-effectiveness should also be considered. Previous studies have confirmed the importance of fidelity in simulation activities. However, no consistent conclusions have been made regarding which fidelity level is best [27]. Thus, which fidelity levels are more efficient in different populations still requires exploration in future studies.

The present study has several strengths. First, a course combining theory, practice, and multiple nursing courses was conducted taken into account the students’ characteristics, which demonstrated the comprehensive concept of the course. Also, scenario simulations were applied in an RN-BSN education and confirmed the applicability and acceptance of simulations among RNs. Moreover, this study revealed that nurses with more work experience rated the scenario simulation training highly.

Despite the above strengths, the present study still has some limitations. First, a group of 4 to 6 students is ideal [27], but this study had 7 to 8 students in each group, with some remaining without “active” roles and had to be observers. This resulted in less involvement in the simulation. Second, this study focused on measuring the students’ evaluation of the simulation module, but not on the students’ competencies. In future studies, we will add indicators to evaluate students’ competencies.

## Conclusions

This comprehensive nursing skills course, integrated into the core nursing curriculum, used scenario simulation, which was demonstrated to improve students’ satisfaction and self-confidence. Students with different work experience times rated the simulation module practice and design highly, which showed that simulations are widely applicable for RN-BSN students, not just for pre-licensed students. Future researches should fully consider the characteristics and needs of students, especially of nurses with work experience. Also, a survey conducted before class might help design the scenario simulation with more targeted content and difficulty. Additionally, recording students’ performance in simulations and evaluating their competence from multiple perspectives are also required.

## Data Availability

The datasets generated during and analyzed during the current study are not publicly available due to the access is bound by the ethical agreements in the project, and out of respect for participants’ privacy but are available from the corresponding author on reasonable request.

## Abbreviations

NAM: National Academy of Medicine
RN: Registered nurses
BSN: Bachelor of Science in Nursing
ADN: Associated Degree in Nursing
S-CVI: Scale-level Content Validity Index.
